# IMPORTANCE OF ASSESSING FOR POSTTRAUMATIC STRESS DISORDER IN OLDER HISPANICS

**DOI:** 10.1093/geroni/igae098.1585

**Published:** 2024-12-31

**Authors:** Sandy Alcantar

**Affiliations:** Los Angeles County Department of Mental Health, Los Angeles, California, United States

## Abstract

Hispanic older adults are at a higher risk of being diagnosed with Alzheimer’s disease or Dementia versus post-traumatic stress disorder due to various factors, one lacking assessment. There are significant factors associated with aging and PTSD in the Hispanic population that must continue to be explored; these include, but are not limited to, a lack of collaboration with community providers, linguistics, and an understanding of assessing for histories of post-traumatic stress disorder. Providers need to evaluate for trauma in Hispanics at this stage of life, especially for those who suffered trauma in their country of origin and post-immigration to the United States. The objective is to reduce misdiagnoses of Alzheimer’s Disease/Dementia in Hispanics by developing and implementing a solid community partner collaboration and not disregarding assessing for post-traumatic stress disorder in a person’s history. As providers, we must communicate effectively in an interdisciplinary approach to review findings during assessments and promote structural social changes, such as education, addressing assessment practices, using effective evidence-based techniques, and reviewing treatment recommendations periodically.

